# Public perception of animal welfare in Iran

**DOI:** 10.1017/awf.2025.10054

**Published:** 2025-12-17

**Authors:** Azadeh S Jalali-Motahari, Mandy B A Paterson, Amin Azadian, Michelle Sinclair

**Affiliations:** 1 Veterinary Health Care, Independent Scholar, Sari, Mazandaran, Iran; 2 School of Veterinary Sciences, University of Queensland, Gatton, Australia; 3 Animal Welfare Program, Faculty of Land and Food Systems, University of British Columbia, Vancouver, BC, Canada; 4 A World of Good Initiative Inc, Santa Fe, New Mexico, USA

**Keywords:** Animal welfare, attitudes, Halal, Iran, perceptions, Persia

## Abstract

While animal welfare is a growing global concern, there has been very little research into how it is understood in Iran. Cultural, religious, and legal factors influence attitudes and practices in ways not addressed by existing research. This study provides culturally grounded insights for improvement of animal welfare in Iran. Utilising a validated survey tool, we investigated the attitudes of Iranians toward the welfare of farmed, companion, and wild animals. A total of 325 responses were collected. The findings indicate that animal welfare is considered important to Iranians, with the majority expressing interest in improving the welfare practices. Despite varying degrees of familiarity with different animal species, there was a consensus on the importance of enacting laws to protect animal welfare. Most participants agreed that chickens feel pain (92.9%) and emotions (79%), whereas fewer attributed these capacities to fish, with 63.6% acknowledging pain and 59.5% acknowledging emotions. Furthermore, most of the participants agreed that animals should not endure pain in the slaughter process (97.8% agreement). While the majority of participants agreed that pre-slaughter stunning was better for the animals (78.7%), only 51.7% agreed that they would prefer to eat meat from animals that had been stunned; reflecting the traditionally held views regarding the role of stunning in Halal meat production. The results of the current study support previous findings suggesting that concern for animals may be a universal human inclination, although, in Iran, attitudes towards specific species and agricultural practices are also shaped by religious perspectives.

## Introduction

Formerly known as Mesopotamia and the seat of power of the ancient Persian Empire, Iran is often referred to as the cradle of civilisation (Kramer 1969). The older a country’s civilisation, the more complex the contemporary attitudes toward various aspects of modern life become. This is understood to reflect the complex interaction of religion, history, and cultural values which evolve over time (Emeng & Okafor 2022 ; Riviș-Tipei 2023).

The concept of animal welfare in Iran is embedded in this long cultural legacy and historical traditions. In ancient Iran, a long-standing culture of respecting animals was based upon a holistic world view, where success in life depended on harmony with all parts of the world, from water, soil, and air to plants, animals, and humans (Ebrahimi & Jafaritabar 2021). With an estimated 99.4% of Iran’s 86.8 million population being Muslim (98%; Association of Religion Data Archives, n.d.), these principles are likely to still be highly relevant to the Iranian culture and attitude towards animals.

People’s attitudes, views, empathy, and prior experiences with animals can strongly influence how they interact with and respond to animals, and how they think humans should treat animals (Prato-Previde *et al.* 2022). In turn, these views are influenced by broader social, cultural, and situational contexts, such as civic and spiritual culture (Landim *et al.* 2024), crisis situations (Oosthuizen *et al.* 2023), economic recession (Weng & Hart 2012), moral awareness (Browning & Veit 2023), trauma (Ladny & Meyer 2019 ), geography and geopolitical culture (Sinclair & Phillips 2017; Sinclair *et al.* 2022a, 2023), age (Li *et al.* 2018; Carnovale *et al.* 2022), and gender (Serpell 2004). Supporting animal welfare can have a profound effect upon the quality of relationships between humans and animals (Verga & Michelazzi 2010; Escobar-Ibarra *et al.* 2021; Orihuela 2021). It also improves practical strategies for animal management, especially within the animal production context (Boyle *et al.* 2022), with the goal of creating sustainable, ethical, and socially accepted animal practices. Regardless of what shapes individual perspectives, ignoring animals’ capacity to have rich emotional lives requiring an optimal level of welfare reduces opportunities to better understand animals with whom we share this planet (Bekoff 2000).

The aim of this research is to evaluate the current attitude of the general public in Iran towards the welfare of animals. Findings from this study provide insight into the level of public awareness and concern regarding various aspects of animal life. There is very limited empirical research regarding public attitudes towards the quality of life experienced by animals in Iran, and almost none based on qualitative interviews. Such data are crucial for assessing the extent to which the Iranian community is aware of, and understands, diverse perspectives on animal welfare and quality of life across species. This knowledge can then help guide policy-makers, educators, and advocacy groups in developing initiatives that are both culturally relevant and effective in raising welfare standards. Novel cross-cultural comparisons will also allow for the identification of universal human perceptions, in addition to those that are unique to Iran. To the authors’ knowledge, this is the first research of this nature to be conducted in Iran.

## Materials and methods

### Research ethics

Research ethics approval for this study was obtained from the University of Queensland, Australia (Ref no: 2020HE002752). Due to the internationally collaborative nature of this study, and the current global relations climate between Iran, USA and Australia, supplementary approvals were sought and obtained in the course of obtaining ethical approval and proceeding with data collection.

### Research tool

This study adopted a validated research tool previously used to collect data on individual perceptions of animals and their welfare in various cultural contexts (Sinclair *et al.* 2022a,b), and applied it to the Iranian context.

The original research tool consists of 25 items, and we added an extra item on wildlife welfare for this research. There were four extra demographic questions (identified gender, age group, education level, and religion). The research items were designed to assess participants’ knowledge of the meaning of animal welfare, their opinion regarding the importance of animal welfare (farm, companion and wild animal), opinion about animals’ ability to experience pain and emotions (chickens and fish), and thoughts about animal slaughter processes. The survey mainly asked participants to indicate their level of agreement with statements on a 7-point Likert scale (1 – strongly disagree, 4 – neither agree nor disagree, 7 – strongly agree), or to indicate the level of importance they attributed to the welfare of various species (1 – extremely unimportant, 4 – I don’t have an opinion/don’t know the species, 7 – extremely important). There were also a few multiple-choice questions.

Participants were initially provided with the following definition of animal welfare:

*“‘The welfare of animals refers to how well an animal is coping with the conditions in which it lives. An animal has good welfare if its needs are being met, and hence it is healthy, comfortable, well-nourished, safe, able to express important behaviors and not suffering from unpleasant states such as pain, fear, and distress”* – Adapted from World Organisation for Animal Health (OIE 2016).

The tool was translated into Farsi, pilot-tested and revised in consultation with research colleagues to ensure it was appropriate for the Iranian context.

### Data collection

**Figure 1.**
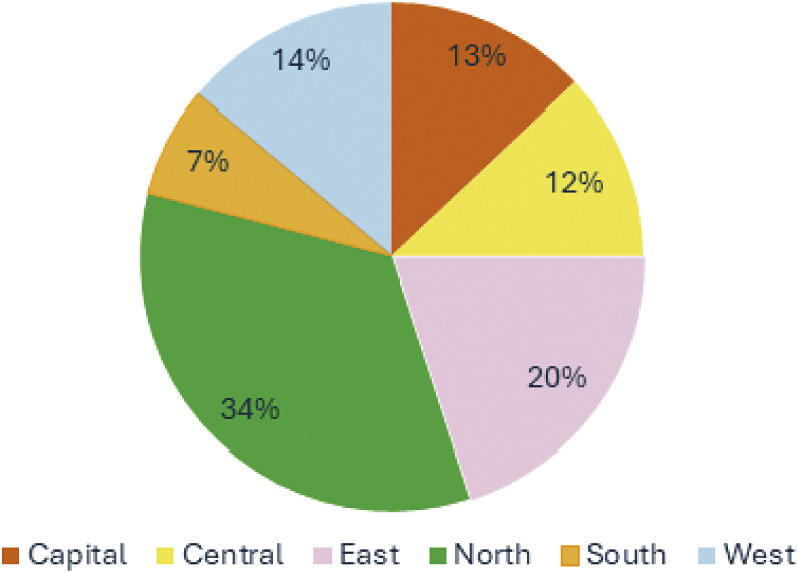
Geographical distribution of survey participants (n = 325) across Iran’s north, south, east, west, central and capital regions.

Data collection took place between January and May 2024. The survey was conducted at an intercity bus station in northern Iran, through which people from all over the country travel. The use of this site for participation recruitment ensured participants were geographically diverse (Figure 1). Although the research tool was hosted online, the primary method of data collection was face-to-face interviews with people randomly selected in public spaces. The data collector (ASJM) was a local researcher fluent in Farsi who was familiar with animal welfare concepts. Prospective participants were randomly selected and approached by ASJM and asked if they would be willing to complete a five-minute survey regarding their opinions about animals for an international academic study. If they agreed ASJM ensured participants were over 18 and that they identified themselves as residents of Iran. If they did not meet the criteria, they were thanked for their time, and data collection ceased. If they met the criteria and agreed to participate, they were informed that they could withdraw from the survey at any time. They were presented with a statement of consent prior to commencement, and their continuation in the study was contingent upon obtaining their consent. ASJM then presented each item to the participants (in Farsi) and entered their responses into the online survey tool, where it was stored anonymously. While this method of collection was resource-intensive, it was adopted to reduce the bias of self-selection which might favour animal-leaning participants. This method had been validated using the same research tool in previous studies conducted in Australia, Bangladesh, Chile. The People’s Republic of China (henceforth China), India, Malaysia, Nigeria, Pakistan, Philippines, Sudan and Thailand (Sinclair *et al.* 2022a,b, 2023).

### Data analysis

The data were initially collated, organised and cleaned by removing incomplete datasets. The data were then imported into Microsoft® SQL Server and Microsoft® Excel for further cleaning, and IBM® SPSS (SPSS® 29.0) and Minitab® (Minitab® Statistical Software) were used to obtain descriptive statistics for the demographic and research items. Based on the Likert scale (1–7), all attitudinal questions were assessed for their means to approximate the magnitude of agreement with statement items, or their associated importance regarding species-specific animal welfare perceptions (Norman 2010). Percentages of agreement (General Agreement Percentage [GAP]) were calculated by identifying and quantifying the number of participants who expressed some level of agreement (values 5, 6 or 7), compared to those who expressed disagreement (values 1, 2 or 3) or neutrality (value 4).

## Results

### Participants

A total of 325 individuals participated in this study and completed the research tool across 63 geographically diverse cities and towns in Iran (north, east, west, south, central, and capital regions). The demographic distribution of respondents across the country is presented in Figure 1. The sample was closely split by gender (51.9% male, 48.1% female) and most participants were aged between 18 and 49 years (83.1%). Participants with a university education were overrepresented compared to the general population; however, this has been a consistent trend observed in other countries where this method was adopted.

### Core perceptions

Most participants (95.6%) agreed that the welfare of farmed animals in Iran was important to them (Table 1). In addition, 80.3 and 98.4% of participants agreed that the welfare of companion animals and wild animals, respectively, matters to them (Table 1).

**Table 1.** Participant (n = 325) responses to 5 core perception items on a Likert scale from 1–7 where 1 is strongly disagree and 7 is strongly agree

Core perception items	General agreement (% with score 5, 6 and 7)	Mean Likert Score	Standard deviation (SD)
The welfare of farmed animals in Iran matters to me	95.6	6.1	0.8
The welfare of dogs and cats (Companion Animals) in Iran matters to me	80.3	5.4	1.6
The welfare of wild animals in Iran matters to me	98.4	6.4	0.7
If I could afford it, I would pay more to buy products that are kinder to animals	88.3	5.8	1.2
I think it is important to have laws that protect the welfare of animals in Iran	97.2	6.2	0.7

Eighty-eight percent of respondents indicated that if financially feasible, they would choose to spend more on products that adhere to higher standards of animal welfare (Table 1). And most participants (97.2%) agreed that it was important to have laws that protect the welfare of animals in their country (Table 1).

### ‘Importance’ of animal welfare by species

**Figure 2.**
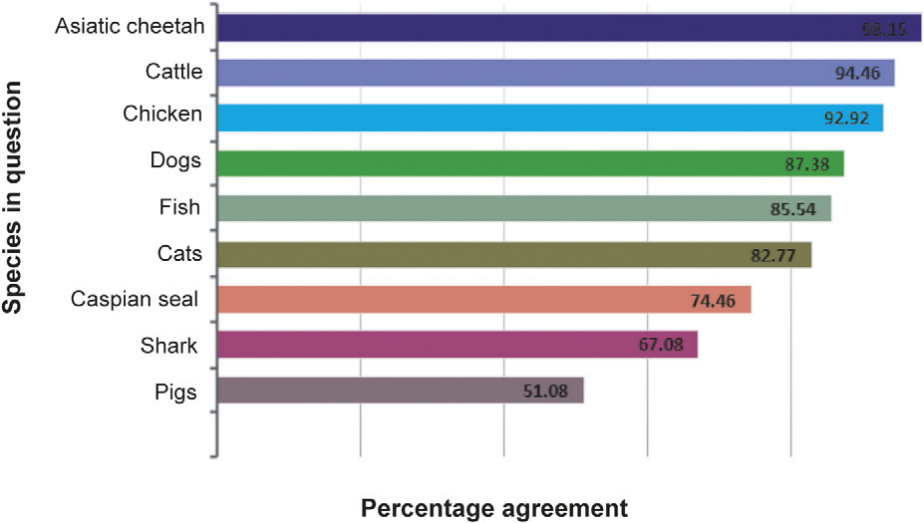
Participant (n = 325) ranking of the perceived importance of animal welfare for various species in Iran. The welfare of each assessed species is ranked according to the percentage agreement achieved.

Overall, the welfare of various species assessed in this study was considered important to varying degrees, with sharks and pigs ranking the lowest. For our participants, the welfare of humans was considered the most important among the assessed species (GAP = 99.3%). The other assessed species were ranked in order of the importance of their welfare as follows (ranked by GAP): Asiatic cheetah (*Acinonyx jubatus venaticus*; 98.1%; n = 319); cattle (94.4%; n = 307); chickens (92.2%; n = 302); dogs (87.3%; n = 284); fish (85.5%; n = 278); cats (82.7%; n = 269); Caspian seals (*Pusa capsica*; 75.3%; n = 242); sharks (67%; n = 218); and, lastly, pigs (51.9%; n = 166) (Figure 2).

### The experiences of chicken and fish

Participants generally consider that chickens feel pain more than fish (92.9 vs 63.6%), that chickens also feel emotions more than fish (79 vs 59.5%), and that chickens need room to explore (96.6%; Table 2).

**Table 2.** Participant (n = 325) levels of agreement with statements about the ability of chickens and fish to experience pain and emotions on a Likert scale from 1 (strongly disagree) to 7 (strongly agree)

Perception of pain and feeling emotions	General agreement (% with score 5, 6 and 7)	Mean Likert Score	Standard deviation (SD)
Chickens can feel pain	92.9	6.2	0.8
Chickens experience emotions	79.0	5.7	1.2
Chickens need room to explore and exercise	96.6	6.3	0.7
Fish can feel pain	63.6	5.2	1.4
Fish experience emotions	59.5	5.0	1.5

### Perceptions toward animal slaughter

Seventy-two percent of participants disagreed with the statement “*I am comfortable watching an animal slaughtered for meat*”, 25.2% agreed and 2% were indifferent (Table 3). At the same time, 97.8% agreed with the statement “*It matters to me that the animals do not suffer during slaughter”.* However, only 48.9% stated that they were aware of the slaughter process, 32% stated they were aware to some extent, and 19.1% stated they were not aware of the slaughtering process at all.

**Table 3.** Participant (n = 325) responses to statements regarding animal slaughter on a Likert scale from 1 (strongly disagree) to 7 (strongly agree)

Perceptions toward animal slaughter	General agreement (% with score 5, 6 and 7)	Mean Likert Score	Standard deviation (SD)
I am comfortable watching an animal slaughtered for meat	25.2	2.5	2.1
It matters to me that the animals do not suffer during slaughter	97.8	6.4	0.7
It matters to me how the meat is produced	97.2	6.2	0.7
I think that stunning an animal unconscious immediately prior to slaughter is better for the animal	78.7	5.6	1.5
I think stunning the animal unconscious will reduce the taste of the meat	30.7	4.0	1.5
I would prefer to eat meat from animals that are made stunned unconscious prior to slaughter	51.7	4.7	1.6

When considering slaughtering and meat production, 97.2% agreed with the statement *“It matters to me how the meat is produced”.* Similarly, 78.7% agreed with the statement *“I think that stunning an animal unconscious in the moments before they are slaughtered is better for the animal”* and 10.7% stated that they did not know. However, the participants were ambivalent about the statement “*I think stunning an animal unconscious will reduce the taste of the meat*” with 30.7% agreeing but not strongly, 42.7% declaring that they did not know, and just 26.1% disagreeing with the statement. In total, 51.7% stated they prefer to consume meat from animals that are stunned before slaughter (Table 3).

### Perceptions toward eggs and egg production systems

Amongst participants, 97.8% reported eating eggs (Table 4) and 96.6% reported that it matters to them that the chickens producing eggs do not suffer. In response to the statement *“I would prefer to buy eggs from chickens that have not been kept in cages”*, 83.6% agreed, and 13% responded with “I don’t know”. In addition, 65.5% of participants considered that most egg-laying hens in Iran were kept in cages, 25.5% stated that they did not know, and 12% thought that laying hens were not kept in cages.

**Table 4.** Participant (n = 325) responses to statements regarding eggs and egg production systems on a Likert scale from 1 (strongly disagree) to 7 (strongly agree)

Perceptions toward eggs and egg production systems	General agreement (% with score 5, 6 and 7)	Mean Likert Score	Standard deviation (SD)
It matters to me that the chickens producing eggs do not suffer and are healthy.	96.6	6.2	0.7
I would prefer to buy eggs from chickens that have not been kept in cages.	83.6	5.9	1.1

## Discussion

This study assessed the attitudes of Iranian participants toward animals and their welfare using a validated research tool previously used in numerous culturally and geopolitically diverse countries (Sinclair *et al.* 2022a,b, 2023). This allows for an international comparison.

### Farmed animal welfare

The survey clearly demonstrates that the Iranian participants considered the welfare of farmed animals to be very important, which ranks Iran amongst the highest in a comparison with 14 other countries (Sinclair *et al.* 2022a). For example, 95.6% of our participants agreed that farmed animal welfare is important, similar to participants in Chile (96.8%), Pakistan (95.2%), Australia (91.2%), and Brazil (90.2%; Sinclair *et al.* 2022a).

The livestock and poultry industries are important national resources in Iran, providing a significant revenue stream and essential food supplies for the country (Shahrabi-Farahani *et al.* 2024). Approximately 80% of the participants stated that they, at least to some extent, know about the slaughtering process and more than 60% considered that laying hens are being kept in cages.

### Perception of animal pain and feelings (chickens and fish)

The recognition that animals are sentient beings, capable of suffering and experiencing emotions, forms one of the foundations of animal welfare (Le Neindre *et al.* 2017).

Participants expressed differing views regarding the perception of pain and emotions in fish and chickens: 92.2% thought chickens could experience pain and 79% considered chickens capable of experiencing emotions. However, Iranian participants, similar to participants in previous studies in Australia, Bangladesh, Brazil, Chile, China, India, Malaysia, Nigeria, Pakistan, Philippines, Sudan, the UK, and the USA (Sinclair *et al.* 2022a), were less likely to agree that fish experience emotions compared to chickens. A total of 63.6% of our participants considered fish capable of experiencing pain and 59.5% agreed that fish experience emotions.

Furthermore, the participants, as consumers of eggs (97.8%), consider that laying hens need space and room for activities such as roaming and jumping. This view toward laying hens presents a promising opportunity to improve poultry welfare in Iran, particularly by promoting the use of housing systems that extend beyond cages and provide hens with access to littered floors and verandas (Nielsen *et al.* 2023).

### Egg production systems

Prior to the mid-20th century, poultry production in Iran was small-scale, primarily involving indigenous breeds for local consumption. Commercial poultry farming only emerged in Iran in the mid-20th century (Shariatmadari 2019), but it now constitutes a significant and growing portion of livestock farming in the country. Most participants in this study agreed that it mattered to them that egg-producing chickens should not suffer (96.6%). They also preferred to buy eggs produced by hens that are not kept in cages (83.6%). Similar findings have been reported in other countries, including Australia, Brazil, Chile, China, India, Malaysia, Nigeria, Pakistan, Philippines, Sudan, Thailand, the UK, and the USA (but not Bangladesh) (Sinclair *et al.* 2022b).

Despite 83.5% expressing the desire to only buy eggs from uncaged hens, the survey indicated that 25.5% of participants are not familiar with commercial hen housing systems. This notable level of unfamiliarity with hen housing and production systems is consistent with the findings from other countries (Sinclair *et al.* 2022b). Although traditional egg production in villages sees hens grazing on communal pastures (Kamalzadeh *et al.* 2008), the majority of egg production in Iran is from laying hens confined to small cages or occasionally in barns (Kamalzadeh *et al.* 2009); these restricted environments may negatively impact quality of life and hinder laying hens from exhibiting natural behaviours.

### Perceptions of animal slaughter

A total of 72% of participants reported discomfort at the idea of observing animal slaughter for meat production. A similar level of discomfort was also reported by participants in Thailand, India, Australia, Brazil, Chile, and the UK (Sinclair *et al.* 2023). Surprisingly, 25.2% reported feeling comfortable watching slaughter. The Eid al-Adha (the sacrificial slaughter during the completion of the Hajj pilgrimage, which is the fifth pillar of Islam) is a religious practice in some families and may explain why participants from Muslim-majority countries, including Iran, could be more familiar and comfortable with slaughter (Sinclair *et al.* 2023).

The importance of ensuring that the process of slaughter causes minimal suffering to animals was recognised by 97.8% of participants. *The Compliance with OIE Animal Welfare Standards in Slaughterhouses in Tehran Province, Iran: An Introductory Survey*, asserts that the welfare of livestock and farmed animals is important throughout the pre-slaughter and slaughter processes (Khaneghahi Abyaneh *et al.* 2020). To meet the World Organisation of Animal Health Standards for Animal Welfare (standards to which Iran has subscribed), every slaughterhouse should adhere to specific animal welfare directives to ensure high standards of care at all stages of the slaughter process (Khaneghahi Abyaneh *et al.* 2020). The primary way to reduce the suffering of animals during slaughter is to apply stunning — to effectively and instantaneously render the animal unconscious — prior to slaughter, with the animal remaining unconscious until their death (Gregory 2008). While stunning is not yet obligatory in Iran, in cases where it is practiced it must be reversible to ensure both the physical integrity and the Halal status of the meat (Nakyinsige *et al.* 2013). ‘Reversible’ here means the animal, if not slaughtered, would eventually regain consciousness and return to full heath — satisfying the Halal requirement for slaughtered animals to be in full health. Research by Farouk *et al.* (2016), points out that new stunning systems are under development that promise to offer assurance that animals are reversibly unconscious but otherwise in full health at the time of slaughter. In addition to minimising animal distress, the other benefit of inducing unconsciousness in animals during stunning is to reduce movement, allowing for swift and safe slaughter (Bergeaud-Blackler 2007). In line with participants in other countries, a majority of Iranians (97.8%) stated that it mattered to them that animals did not suffer during slaughter, with 78.7% agreeing that it is better for the animals to be unconscious at time of slaughter. However, only 51.7% agreed that they would prefer to eat meat from animals that had been rendered unconscious (stunned), drawing attention to the existence of a significant subset of traditional thinking or uncertainty pertaining to Halal acceptability around stunning.

In Iran, slaughter methods in practice vary between modern and traditional facilities. Electrical stunning is routinely used for sheep in all modern abattoirs in Iran, whereas traditional slaughterhouses typically do not stun animals prior to slaughter (Khanghahi Abyaneh *et al.* 2020). For poultry, water-bath electrical stunning is the most common method in industrial facilities; however, concerns remain regarding its reversibility, particularly if there is a delay between stunning and slaughter or if the voltage is set too high (Riaz *et al.* 2021). Consequently, traditional operations continue to slaughter poultry without stunning.

A significant minority of participants (30.7%) indicated that the application of pre-slaughter stunning negatively impacts the taste of the meat. This is a view shared by minorities in other areas of the world, such as China (Sinclair *et al.* 2019). Scientifically, however, the quality of meat from stunned sheep and cattle has been reported to be similar to that from animals slaughtered without stunning (Gregory 2008). Furthermore, stunning (whether pre- or post-stun) was reported to have a non-significant effect on bleed-out/total blood loss during slaughter, which is also an issue of public concern and could have implications for the hygiene of the meat (Anil *et al.* 2006; Sazili *et al.* 2023).

### Companion animal welfare

While many countries attribute similar levels of importance to companion animal welfare as they do for farmed animals, including Chile (99.2%), Australia (94.4%), Brazil (94%), India (91.8%), Pakistan (92.4%), Thailand (91.3%), and the UK (90.9%) (Sinclair *et al.* 2022a), participants in Iran rated their welfare slightly lower at 80.3%. The reasons for this reduced rating are not certain, but societal and cultural factors potentially play a role. Divergent views regarding the status of dogs among Islamic religious scholars are well-documented and cannot be ignored. Many Muslim cultures, Iran included, do not accept dogs as companion animals sharing their homes and the keeping dogs is discouraged in traditional Islamic jurisprudence due to an interpretation of teachings that designate dogs as ‘unclean.’ However, some contemporary religious scholars assert dogs to be inherently pure, arguing that there is no Quranic or rational justification for declaring them unclean. Furthermore, certain religious texts suggest that, with a degree of leniency, only their saliva may be considered impure. With the advent of updated vaccines and veterinary medicine, even this concern has become largely irrelevant (Mousavi Aghighii 2024). Moreover, caring for dogs involved in activities such as hunting, guarding, and herding livestock is recommended in Islamic teachings. In the Quranic narrative of the *Ashab al-Kahf*, or the *Companions of the Cave*, a dog features prominently thereby redefining the role and status of dogs in Islamic texts (Tlili 2018). To further challenge the religious understanding of dogs, Mohammad Mousavi Aghighii (2024), in his recent national research on ‘*Insight into Dogs in Canon*,’ stated that, due to the similarity between dogs, wolves, foxes, hyenas, and jackals, it was difficult to distinguish what was meant by the word ‘kalb’ in older writings. He asserted that ‘kalb’ (the Arabic word for dog) does not necessarily refer to the modern concept of a ‘dog’. The dissemination of findings like these could help alleviate the unfavourable stigma associated with modern dogs.

Economic constraints, evolving family dynamics, policy gaps, and varying levels of public awareness may all intersect to shape attitudes toward companion animals. In addition, cultural factors — such as the lack of prior exposure to animals, and related emotions like fear, anxiety, or disgust — can strongly affect attitudes. Thus, findings of the current study within this context highlight the potential Iran has for studies into how social, economic, and cultural constraints shape public views on companion animal welfare.

### Welfare of wild animals

The greatest level of concern for welfare in our study was reserved for wild animals (98.4%). Due to its diverse geological formations, climates and soils, Iran is home to enormous biodiversity (Jowkar *et al.* 2022). The promotion of public awareness regarding the impacts of human activities, as well as participation in biodiversity conservation through national strategies, such as broadcasting on the Iranian documentary channel, conducting special training programmes for instructors, educators, and local communities, and establishing environmental and ecological groups, might have significantly influenced public understanding of wildlife conservation and welfare issues (Mohammadian *et al.* 2015). Iranian conservation NGOs are taking an active role in promoting awareness and preserving wildlife biodiversity in Iran (Jowkar *et al.* 2022). Considering these findings, such initiatives would appear effective in raising awareness of the plight of wild animals.

### Species perceptions

The perceived importance of the welfare of various animal species was assessed in this study. Humans and Asiatic cheetahs were ranked the highest, followed by cows and chickens, with the Caspian seal, sharks, and pigs ranked last. As with previous studies using this research tool, Iran, as a predominately Muslim nation, saw reduced importance placed on the welfare of pigs. This perspective may be exacerbated somewhat by reports of wild pigs attacking farms in some rural areas, causing destruction. Sharks, surprisingly perhaps, were ranked alongside pigs in terms of welfare concern. More than 60 species of shark have been recorded in the waters of the Persian Gulf and the Sea of Oman with attacks in the Persian Gulf viewed as a potential threat to divers and sailors (Morshedi *et al.* 2020). Conservation efforts are constrained by public fear (galeophobia) and misunderstanding (Casola *et al.* 2022). Despite the statistical rarity of shark attacks on humans, this risk however unlikely can cause widespread fear, aided by media sensationalism (Le Busque *et al.* 2021).

At the opposite end of the scale, the Asiatic cheetah was ranked the highest in importance. Once covering vast areas of the Arabian Peninsula, from Southeast Asia to India, this feline species is the most endangered subspecies of cheetah, now restricted to a small population in Iran (Farhadnia *et al.* 2017). Considerable effort has recently been invested into cheetah conservation, but continued action is essential to save the species from extinction (Farhadnia *et al.* 2019). The national pride associated with the cheetah’s presence in Iran may explain the finding in this study, where 98.1% of participants stated that they care about the Asiatic cheetah. Conservation efforts can be spearheaded through the use of mass media, including TV, radio, newspapers, and social media, which play a unique role in conveying information, shaping public opinion, and influencing policy (Gore & Knuth 2009; Nanni *et al.* 2020). The media environment has become the main platform for the public to advocate for animal protection (Geng 2023). In this case, media-driven awareness has turned the Asiatic cheetah into a national symbol, with its image emblazoned on the Iranian airline fleet and the national football team regalia (Tehran Times 2015).

### Animal protection laws and higher welfare animal products

Having laws that adequately protect a wide range of animals, including companion animals, farm animals and wildlife in Iran is likely to be welcomed. Most of our participants strongly agreed that laws to protect animals were important (97.2%). Compared to findings from 14 other countries, Iranian participants were second only to participants in Chile (98.4%). Agreement on willingness to pay more for products that are kinder to animals, should they be able to afford it, was also reasonably high amongst our participants at 88.3%. It seems clear that Iranians could respond well to a moderately priced, higher-welfare market. The public desire for laws focused on animal welfare, and their preference for purchasing from places that provide better conditions for animals, indicate a significant tendency in societal priorities, reflecting the country’s desire for progress in this area.

### Study limitations

Although efforts were made to avoid sampling from a specific demographic group; asking people travelling on buses on long routes favoured the younger segment of society. Generalisation from our results can only be made with care. Other limitations within this study are common to survey methodologies. The data collected rely upon self-reporting, which can be impacted by social desirability bias and other biases (Grimm 2010). For instance, survey respondents may avoid extreme responses, potentially influencing the findings. The authors also acknowledge that interview participants were randomly selected from among travellers at the bus terminal representing various geographical regions however, no statistical analysis by region was conducted. A further potential limitation of this study is the possibility of interviewer influence on participant responses. As data were collected via in-person interviews, it is possible that participants may have adjusted their answers, consciously or subconsciously, due to the presence of the researcher. This could have contributed to an increase in reported awareness or positive attitudes toward animal welfare between the beginning and end of the survey. This effect is related to, but distinct from, social desirability bias and should be considered when interpreting the results. Future studies could reduce this potential bias by employing fully anonymous or self-administered data collection methods.

The authors also wish to draw attention to the diversity of language; although the translations were carried out carefully and a definition of animal welfare was provided, connotations of specific terms may differ across regions. For example, ‘animal welfare’, ‘emotions’, and ‘pain’ may translate into generic or pre-existing concepts in local language, which can carry slightly different meanings.

### Animal welfare implications

This study suggests that the welfare of farmed, companion, and wild animals is highly valued in Iran, alongside support for legal protection and ethical slaughter practices. These findings highlight an opportunity to develop welfare-focused policy and awareness. Cross-cultural similarities also support global advocacy, while cultural differences, such as religious slaughter norms, call for localised welfare strategies that respect tradition. Policy-makers and stakeholders can use these data to guide interventions and outreach.

## Conclusion

This research represents the first comprehensive investigation into public attitudes toward animal welfare in Iran. The study reveals that the Iranian participants hold complex views regarding animal welfare, influenced by cultural, religious, and economic factors. The majority of participants emphasised the importance of welfare for farmed animals, companion animals, and wildlife, and raised the need for laws to safeguard animal welfare. In Iran, as in other countries, factors such as taste, personal interest, experiences (fear or affinity), utility/benefit to people (functional or non-functional), knowledge, media attention, and religious attitudes can influence individual feelings of amity or enmity toward animals, or particular species. These results suggest a broad recognition of the importance of animal welfare among the Iranian public, which lays a strong foundation for further advancement in this area. The findings of this study indicate that animal welfare is a priority for the people of Iran, presenting an opportunity for the country to develop programmes focused upon education and training in this field. It is essential therefore to establish more policies and laws to raise animal welfare standards in the country.

These results may serve as a foundation for further studies that take into account economic conditions, social values, and scientific perspectives. Such studies could be conducted in academic Centres dedicated specifically to the study of animal welfare and behaviour, contributing to policy development, planning, and identifying target audiences for animal welfare initiatives. The level of public knowledge regarding commercial farming practices, attitudes toward the treatment of companion animals, and wildlife conservation and welfare can drive society toward upholding higher standards of animal welfare.
